# Physical Exercise Induces Immunoregulation of TREG, M2, and pDCs in a Lung Allergic Inflammation Model

**DOI:** 10.3389/fimmu.2019.00854

**Published:** 2019-05-16

**Authors:** Paula Fernandes, Luana de Mendonça Oliveira, Thayse Regina Brüggemann, Maria Notomi Sato, Clarice Rosa Olivo, Fernanda Magalhães Arantes-Costa

**Affiliations:** ^1^Laboratory of Experimental Therapeutics LIM20, Department of Medicine, School of Medicine, University of São Paulo, São Paulo, Brazil; ^2^Laboratory of Medical Investigation LIM56, Division of Clinical Dermatology, School of Medicine, University of São Paulo, São Paulo, Brazil; ^3^Pulmonary and Critical Care Medicine Division, Department of Medicine, Brigham and Women's Hospital and Harvard Medical School, Boston, MA, United States; ^4^University City of São Paulo (UNICID)/Institute of Medical Assistance to the State Public Servant (IAMSPE), São Paulo, Brazil; ^5^Department of Medicine, Center of Development of Medical Education, CEDEM, School of Medicine, University of São Paulo, São Paulo, Brazil

**Keywords:** asthma, physical exercise, immunoregulation, Treg, M2, dendritic cell

## Abstract

The benefits of moderate aerobic physical exercise for allergic asthma are well-known, particularly that of the anti-inflammatory effect that occurs by reducing Th2 responses and lung remodeling. However, the mechanisms of this immunoregulation are still under investigation. In this study, we investigated the possible immunoregulatory mechanisms of lung inflammation induced by moderate aerobic exercise in an experimental asthma model. BALB/c mice were distributed into Control, Exercise (EX), OVA, and OEX groups. OVA and OEX groups were sensitized with ovalbumin (OVA) on days 0, 14, 21, 28, and 42 and were challenged with OVA aerosol three times a week from days 21 to 51. The EX and OEX groups underwent moderate aerobic physical exercise from days 21 to 51 (5 d/w, 1 h/d). The mice were euthanized on day 52. We evaluated pulmonary cytokine production, serum immunoglobulin levels, and the inflammatory cell profile in lung and mediastinal lymph nodes. OVA mice showed increased expression of IL-4, IL-6, IL-10, and TGF-β and decreased macrophage type 2 (M2) recruitment. Physical exercise did not affect the increased antibody production of IgG2a, IgG1, or IgE induced by OVA. Of note, physical exercise alone markedly increased production of anti-inflammatory cytokines such as IL-10 and TGF-β. Physical exercise in OVA-mice also increased the recruitment of M2 in the lungs, as well as the influx and activation of regulatory T cells (Tregs) and CD4 and CD8 lymphocytes. In the draining lymph nodes, it was also observed that physical exercise increased the activation of CD4 T cells, regardless of the presence of OVA. Notably, physical exercise decreased common dendritic cells' (cDCs; pro-inflammatory) expression of co-stimulatory molecules such as CD80, CD86, and ICOSL in the draining lymph nodes, as well as increased ICOSL in plasmacytoid dendritic cells (pDCs; anti-inflammatory). Together, these findings show that physical exercise modulates pulmonary allergic inflammation by increasing Treg and M2 recruitment, as well as pDCs activation, which leads to an increase in anti-inflammatory cytokines and a decrease in pro-inflammatory cells and mediators.

## Introduction

Asthma is a heterogeneous disease characterized by the chronic inflammation of the airways and variable remodeling (1). It is defined by a history of respiratory symptoms, such as wheezing, difficulty breathing, chest tightness, and cough that varies in time and intensity, along with a variable airflow limitation (1, 2). Aerobic exercise has been used in several rehabilitation programs for asthmatic patients, resulting in decreased dyspnea, airway hyperresponsiveness, the induction of bronchospasm and even corticosteroid use. In addition, these patients demonstrate improvements in aerobic capacity and quality of life (3, 4).

Some authors have already shown that aerobic physical exercise may decrease allergic lung inflammation in sensitized animals and have suggested that this reduction may occur by inhibition of nuclear factor activation (NF-κB) or by the increased expression of anti-inflammatory cytokines such as IL1-ra and IL-10 (3, 5, 6). Mackenzie et al. (7) have shown that animals sensitized to ovalbumin (OVA) and submitted to an experimental exercise protocol demonstrate reduced IL-4, IL-5, and IL-13 levels in the mediastinal lymph nodes. Furthermore, this same study demonstrated that physical exercise inhibits maturation and the activation of dendritic cells, indicating that the decrease in Th2 response in sensitized and trained animals may be due to some interference in the maturation of the OVA antigen presenting cell during the exercise (7).

In view of this context, we hypothesize that moderate physical exercise generates greater activation of regulatory cells, thus reducing pulmonary inflammation. To investigate our hypothesis, we conducted this study to investigate the role of some immunomodulatory cells, such as Treg, M2, and pDCs, in the immunomodulation induced by aerobic physical exercise in OVA-sensitized animals.

## Materials and Methods

### Animals

This study was carried out in accordance with the recommendations of the Guide for the Care and Use of Laboratory Animals—NIH (8). The protocol was approved by the Ethics Committee of the School of Medicine of the University of São Paulo (Protocol number 067/16). Male Balb/c mice (6–8 weeks old) were purchased from the University of São Paulo (São Paulo, Brazil) and were divided into four experimental groups (*n* = 10 per group): SAL (non-sensitized and non-trained), EX (non-sensitized and trained), OVA (sensitized and non-trained), and OEX (sensitized and trained).

### Sensitization Protocol

**Figure 1:**
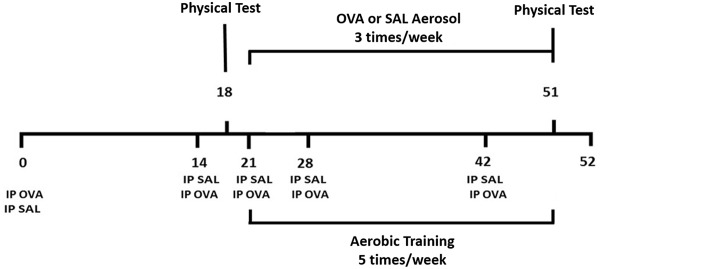
Experimental protocol. Balb/c mice were sensitized with ovalbumin and alumen on days 0, 14, 21, 28, and 42 and were challenged with an aerosol of OVA three times per week, from day 21 until day 51 [OVA and OVA + exercise (OEX) groups]. The EX (exercise) and OEX groups practiced moderate exercise from day 21 until 51. Twenty-four hours after the last challenge the animals were analyzed.

Animals from the OVA and OEX groups were sensitized with five intraperitoneal injections containing 20 μg/mL of OVA (Grade V, Sigma Chemical Co., MO, USA) absorbed in 3 mg/mL of aluminum hydroxide (Pepsamar gel, Snofi-Synthelabo S.A., RJ, Brazil). These animals received a total of 0.2 mL on days 0, 14, 21, 28, and 42. Animals from the SAL and EX groups received five intraperitoneal injections of saline solution (NaCl 0.9%) and aluminum hydroxide on the same days as the OVA and OEX groups. Three times a week, from days 21 to 51, the OVA-sensitized groups were challenged with OVA aerosol at 1%, and non-sensitized groups were aerosolized with saline solution. On day 52, the animals were studied (Figure 1).

### Aerobic Exercise Protocol

The EX and OEX groups underwent an aerobic exercise protocol lasting 5 weeks on a treadmill (KT 400, Imbramed, RS, Brazil), 1 h per day starting on day 21 until day 52 of the experimental protocol. Before starting the training protocol, the mice underwent an exercise test. The test consisted of an initial 5 min running at a speed of 0.2 km/h, and then the speed was gradually increased by 0.1 km/h every 2.5 min until the animal was exhausted. Exhaustion was considered at the time when the animal was unable to remain running even after 10 mechanical stimuli. The training intensity during the experimental protocol was 50% of the average maximum speed reached in the exercise test (Figure 1).

### Specific Antigen-Antibody and Cytokines Analysis

Blood (0.3 mL) was collected from the inferior vena cava of anesthetized animals and diluted with 600 μl of saline solution. Then, it was centrifuged at 3,000 rpm for 10 min at 4°C to obtain serum. The serum was maintained at −70°C until immunoglobulin analysis of OVA-specific (Ig)E (IgE), by Passive Cutaneous Anaphylaxis reaction, as well as IgG1 and IgG2a analysis, by immuno-enzymatic assay (ELISA) according to the manufacturer's instructions (R&D Systems, MN, USA). Both lungs were removed and homogenate adding 600 μL of saline solution, according to manufacture instructions (PowerLyser, MoBio Laboratoties Inc, CA, USA). Interleukin (IL)-4 (IL-4), IL-6, IL-10, and the transforming growth factor (TGF)-β1 levels in the lung homogenate were measured using an ELISA kit according to the manufacturer's instructions (R&D Systems, MN, USA).

### Passive Cutaneous Anaphylaxis (PCA) Reaction

The determination of OVA-specific IgE anaphylactic antibodies was performed by the passive cutaneous anaphylaxis (PCA) reaction, according to the technique described by Mota and Wong (9). Rats were shaved on the back and were sensitized intradermally with serial dilutions of the serum from experimental mice. After a period of 18 to 24 h of sensitization, the animals were challenged intravenously with 0.25% Evans blue containing 500 μg of OVA. The result was determined 30 min after the challenge, measuring the diameter of the positive reaction (blue spots) in the inverted skin of the animals. The probable IgE titers were expressed as the reciprocal of the highest dilution of the serum that resulted in a positive reaction of more than 5 mm in diameter. All tests were done in triplicate, and differences between PCA titers >2 times were considered significant (10, 11).

### Cell Phenotype

Mice (ten per group) were euthanized, and the lungs and mediastinal draining lymph nodes were removed. Lymph nodes were harvested and homogenized using saline solution and a 40 μm cell strainer. The single cell suspension was centrifuged, and the cell pellet was re-suspended in 1 mL of saline for total cell counting. The lungs were cut in small pieces with scissors and incubated in a solution containing collagenase (07 mg/mL, Sigma-Aldrich, MO, USA) and DNAse 1 (30 μg/mL, Sigma-Aldrich, MO, USA) for 30 min at 37°C. After incubation, the single cell suspension was filtered through a 40 μm strainer, and the enzymes were blocked with phosphate buffered saline (PBS) supplemented with fetal bovine serum (FBS) at least three times of the initial volume. Then, the cell suspension was centrifuged at 1,500 rpm for 10 min and re-suspended in 1 mL of saline, centrifuged, and the cell pellet was re-suspended in 1 mL of saline for total cell counting. The cells were stained for cell surface and intracellular markers (Saponine 0.05%, Sigma-Aldrich, MO, USA). The phenotype of the cells was evaluated using the following antibodies: anti-CD3—PERCP CY5.5 (17A2), anti-B220—PERCP (RA3-6B2), anti-MHC-II—PE (M5/114.15.2) or PE Cy7.7 (M5/114.15.2), anti-SIGLECF—PE CF594 (E50-2440), anti-CD11b—PE CY7 (M1/70) or BV605 (M1/70), anti-F4/80—eFluor 450 (BM8), anti-CD11c—FITC (HL3), anti-CD4—V500 (RM4-5), anti-CD69—FITC (H1.2F3), anti-CD8a—APC CY7(53-6.7), anti-CD25—PE CY7 (PC61), anti-FOXP3—V450 (MF23), anti-IL-10—APC (JESS-16E3), anti-latency related peptide (LAP)—PE (TW7-16B4), anti-CD24—PERCP CY5.5 (M1/69), anti-ICOSL—PE (HK5.3), anti-PDL2—APC (TY25), anti-CD86—A700 (GL1), and the viability marker—Texas Red. All antibodies were purchased from BD Biosciences, NJ, USA and eBioscience, SD, USA. A total of 1 × 10^5^ live events were acquired with LSR Fortessa (BD, San Jose, CA, USA) and analyzed with Flow Jo 10.0.6 software (Tre Star, OR, USA). Fluorescence was performed for all antibody panels minus that of one control. The analysis strategy is provided in Supplementary Figures 1–6.

### Statistical Analysis

Statistical differences between experimental groups were detected by analysis of variance (two-way ANOVA) followed by the Holm-Sidak *post hoc* test for multiple comparisons (SigmaStat 2.03, SPSS, Chicago, IL, USA). We considered significant values *p* < 0.05.

## Results

### The Antigenic Exposure Associated With Physical Exercise Maintains Elevated Levels of Antibodies

**Figure 2:**
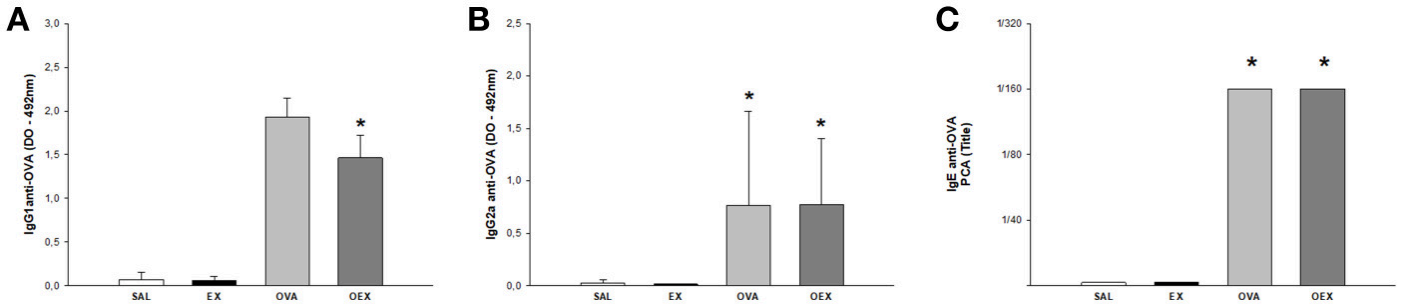
Specific antibodies induced by OVA-sensitization. The results are expressed as the means of the absorbance values (±SE) obtained by ELISA with serum from animals of the various experimental groups **(A)** anti-OVA IgG1 antibodies **(B)** anti-OVA IgG2a. *p = 0.047 compared to the SAL group. **(C)** The mean values of the titer of IgE anti-OVA. *Statistically different values in relation to the control group, SAL or EX.

We analyzed OVA-specific IgG1 and IgG2a levels by ELISA. The results showed an increase of IgG1 and IgG2a levels in ovalbumin-sensitized groups compared to non-sensitized animals. When we evaluated the production and functionality of anti-OVA IgE by PCA, we notice that sensitized animals had higher IgE-related mast cell degranulation titer than 1/160. Similar to IgGs, physical exercise did not modify the intense production of IgE induced by OVA (Figure 2).

### Physical Exercise Reduces Eosinophil Influx to the Lung of Sensitized Animals

**Figure 3:**
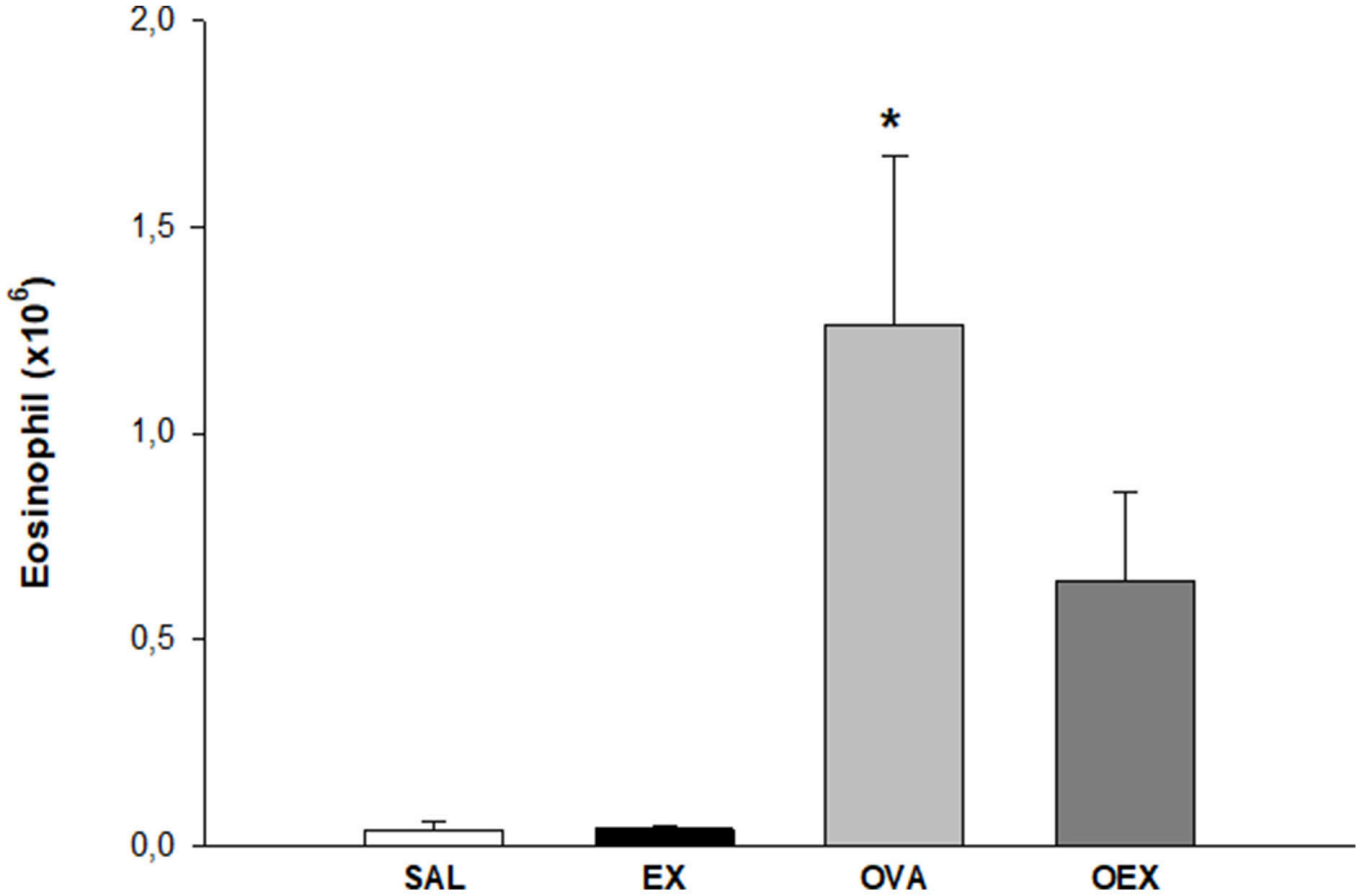
The practice of physical exercise reduces the influx of eosinophils in the lung of OVA-sensitized animals. The mean values of eosinophil counts (±SE) obtained in lung tissue. *p < 0.001 compared to the SAL group. *N* = 10 per group.

The antigen-sensitized group (OVA) showed a high influx of eosinophils in lung tissue that was diminished in OVA-sensitized mice that also underwent aerobic exercise (OEX; Figure 3). In order to explain better anti-inflammatory effect of physical exercise, we measured some cytokines in the lung homogenate.

### Physical Exercise Leads to an Anti-inflammatory Cytokine Release in OVA-Sensitized Animals

**Figure 4:**
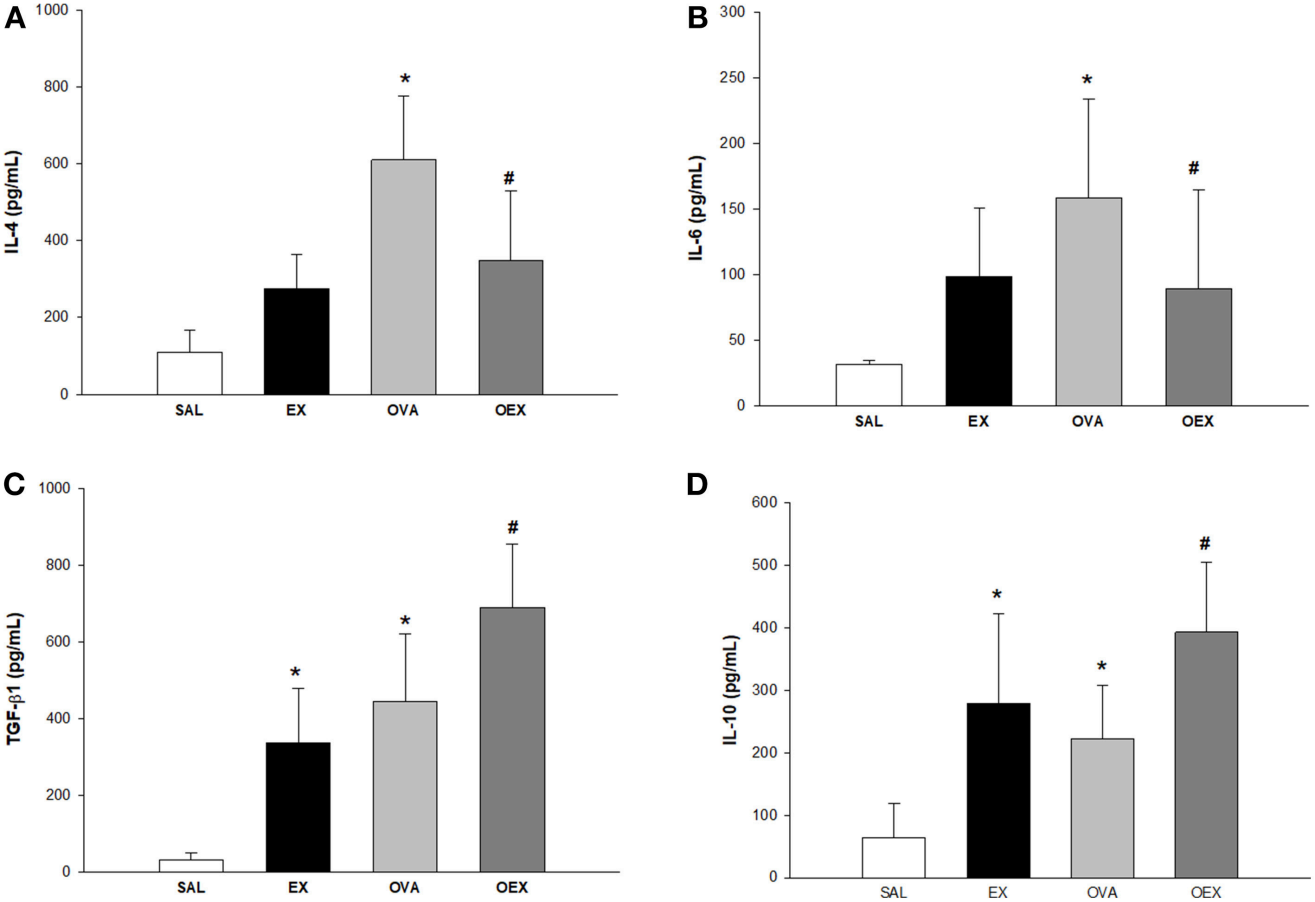
Physical exercise decreases pro-inflammatory cytokines and increases anti-inflammatory cytokines in the lungs of OVA-sensitized animals. The mean values (±SE) obtained in the pulmonary homogenate of **(A)** IL-4 (*p < 0.001 and 0.023, respectively when compared EX or OVA to the SAL group and #*p* = 0.035 compared to the OVA group), **(B)** IL-6 (*p = 0.008 compared to the SAL group and #*p* = 0.035 compared to the OVA group), **(C)** TGF-β and **(D)** IL-10 (*p = 0.001 compared to the SAL group and #*p* = 0.035 compared to the OVA group). *N* = 10 per group.

The quantification of IL-4, IL-6, TGF-β, and IL-10 cytokines from lung homogenate (*n* = 10 per group) demonstrated that sensitization to OVA increased IL-4 and IL-6 levels accompanied by an increase in the levels of anti-inflammatory cytokines IL-10 and TGF-β (Figure 4). In addition, physical exercise reversed this elevation in the OEX group (Figures 4A,B, respectively) whereas increased the production of IL-10 and TGF-β (Figures 4C,D, respectively). We observed that physical exercise alone elevated the levels of IL-4 and also anti-inflammatory cytokines, such as those of TGF-β and IL-10 (Figures 4A,C,D). This anti-inflammatory pattern of cytokines observed in OEX group corroborate with the reduction in eosinophil influx observed in the lung and also with other cellular finds showed above.

### Trained Mice Show an Increased Influx of Lymphocytes and a Greater Activation of These Cells

**Figure 5:**
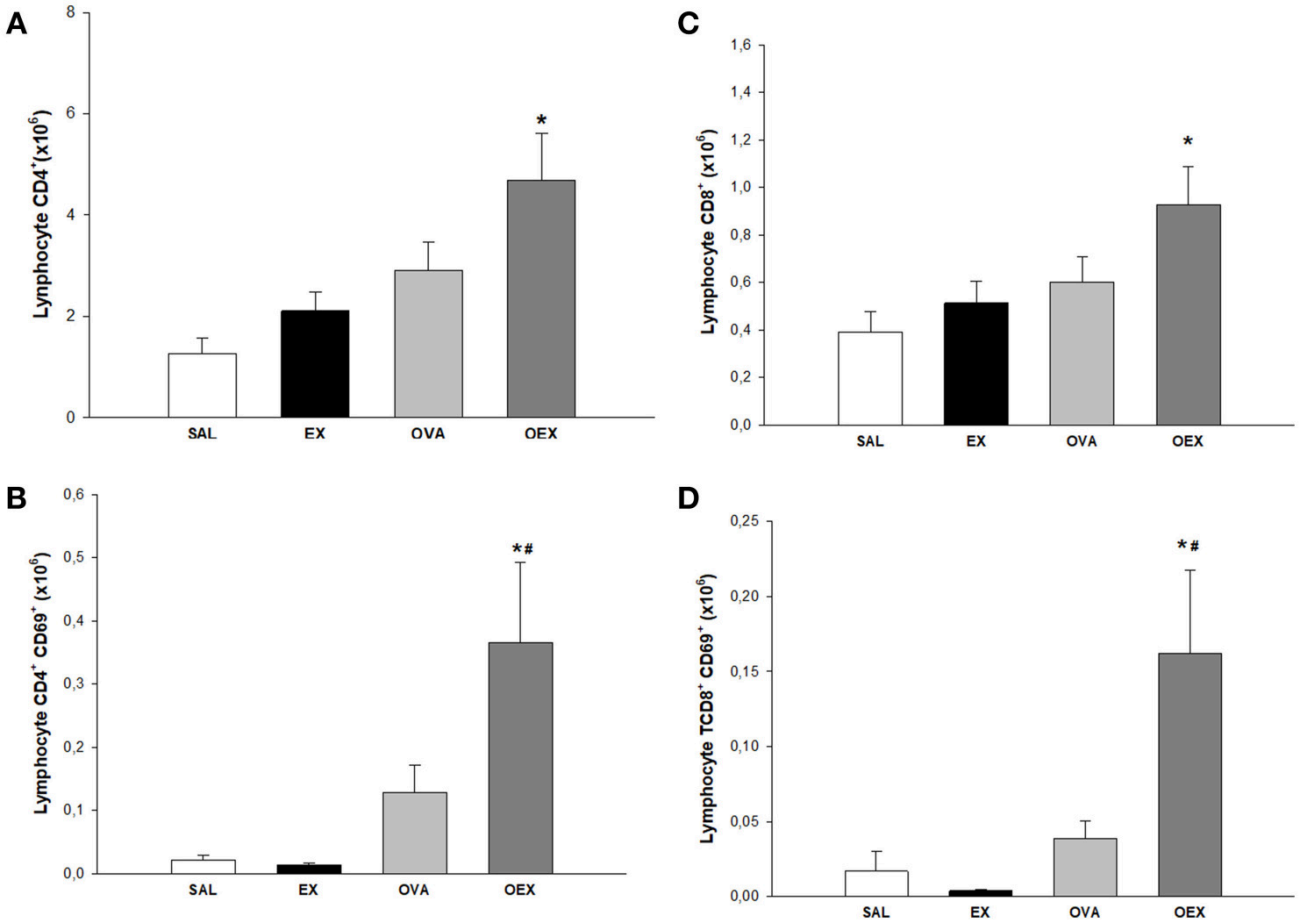
Lymphocytes in lung tissue. **(A)** The mean values of CD4 T cell numbers. *p = 0.01 compared to the SAL group. **(B)** Mean values of CD4 T cells expression of CD69 (±SE). *p = 0.006 compared to EX and #*p* < 0.001 when compared to OVA. **(C)** Mean values of CD8 T cell numbers (±SE). *p = 0.026 compared to the EX group**. (D)** Mean values of CD8 T cells expression of CD69 (±SE). *p < 0.001 compared to the EX group #*p* = 0.004 compared to the OVA group. *N* = 10 per group.

**Figure 6:**
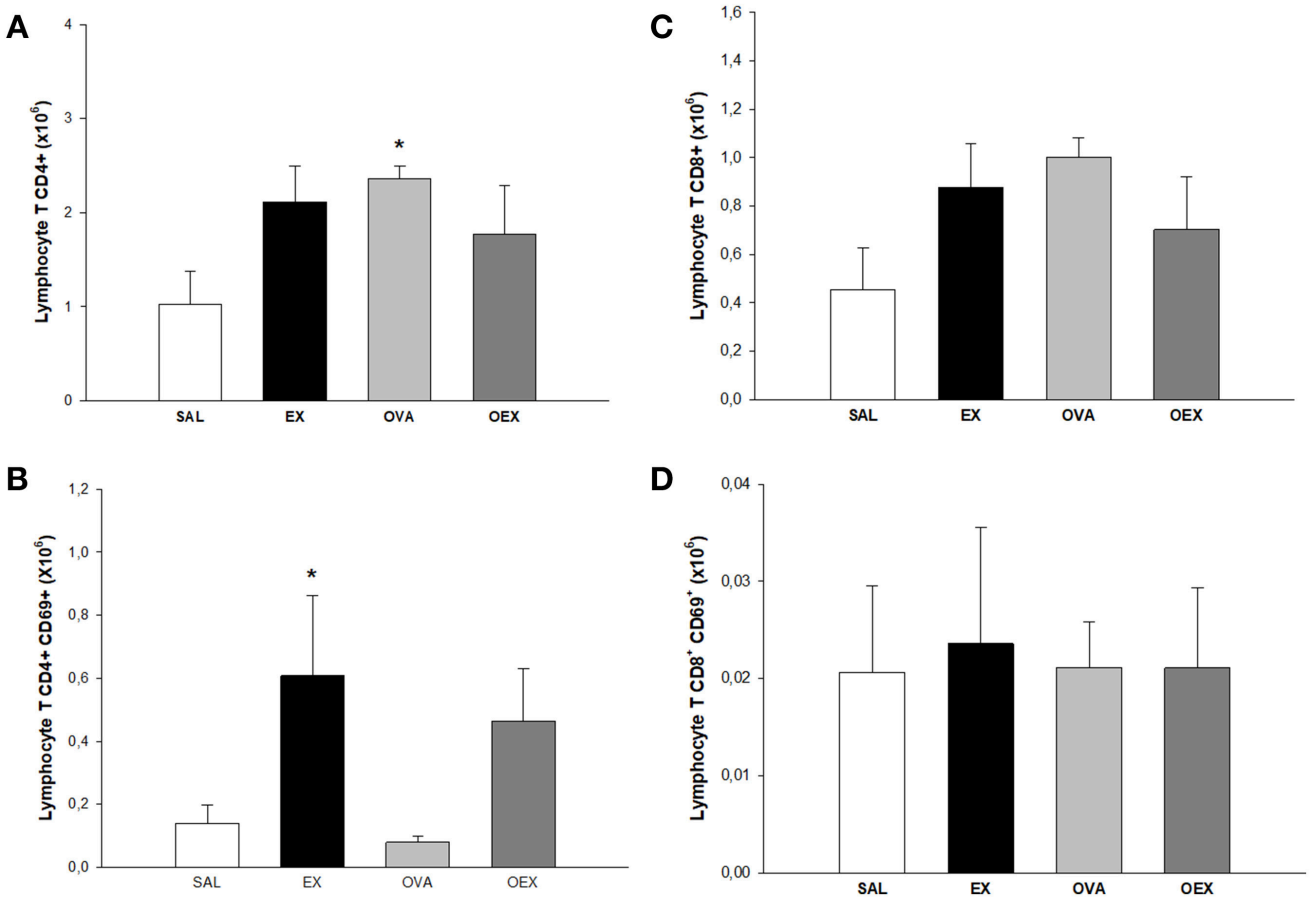
Lymphocytes in lymph nodes. **(A)** The mean values of CD4 T cell numbers. *p = 0.036 compared to the SAL group. **(B)** The mean values of CD4 T cell expression of CD69 (±SE). *p = 0.036 compared to EX. **(C)** The mean values of CD8 T cell numbers (±SE). **(D)** The mean values of CD8 T cell expression of CD69 (±SE). *N* = 10 per group.

To identify the cellular effects of aerobic exercise, we analyzed the inflammatory cell profile in the lungs and mediastinal lymph nodes. Animals that were sensitized and underwent the physical exercise protocol (OEX) had an increase in CD4 and CD8 T cells numbers and activation in the lungs (Figure 5). In the lymph nodes, sensitized animals showed a significant increase in the number of CD4 T cells. Interestingly, we observed that the CD4 T cells in the trained group (EX) showed greater expression of activation markers (Figure 6). This data showed that physical exercise in sensitized mice induced the more pulmonary influx of T cells such as increasing its activation.

### Physical Exercise Induces an Increase of Treg and M2 Regulatory Cells in the Lungs of Sensitized Animals

**Figure 7:**
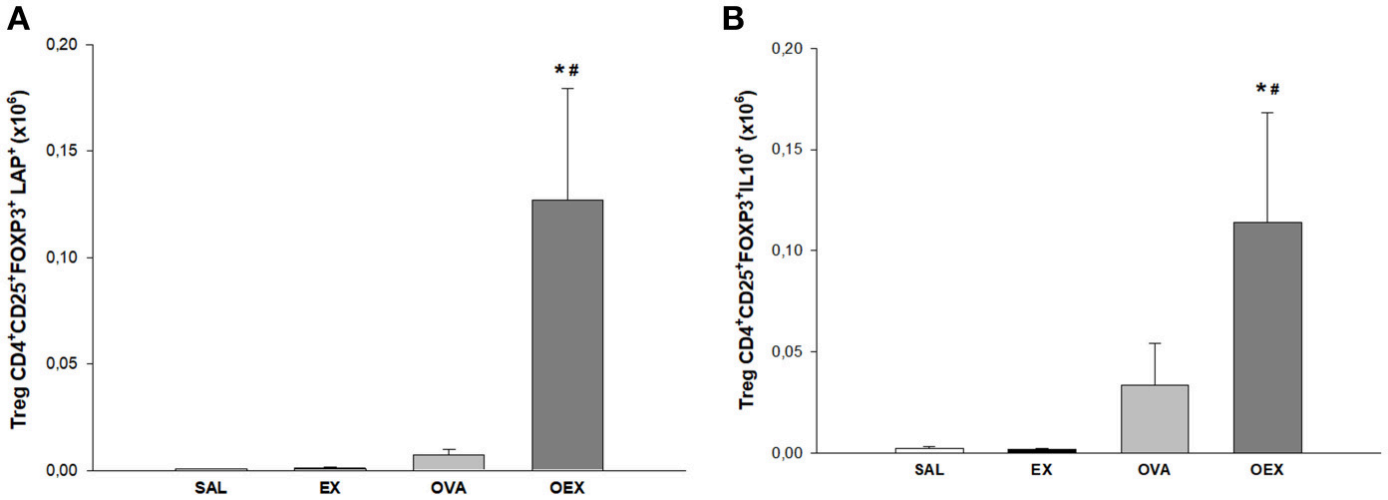
Physical exercise induces higher production of TGF-β and IL-10 by pulmonary Tregs in allergic animals. **(A)** The mean values of TGF-β^+^ (±SE) Tregs obtained from lung tissue. ^*^#*p* < 0.001 compared to the EX group or the OVA group. **(B)** The mean values of IL-10^+^ Tregs (±SE) obtained in lung tissue. ^*^p = 0.011 compared to EX and #*p* = 0.049 to OVA). *N* = 10 per group.

Physical exercise significantly increased activated Tregs (CD4^+^, CD25^+^, FOXP3^+^, LAP^+^) in the lungs of OVA-sensitized and challenged mice. These data suggest that Tregs, which are the main source for TGF-β and IL-10 (Figure 7) are responsible for the local counter regulation of OVA allergen-driven inflammation induced by physical exercise.

**Figure 8:**
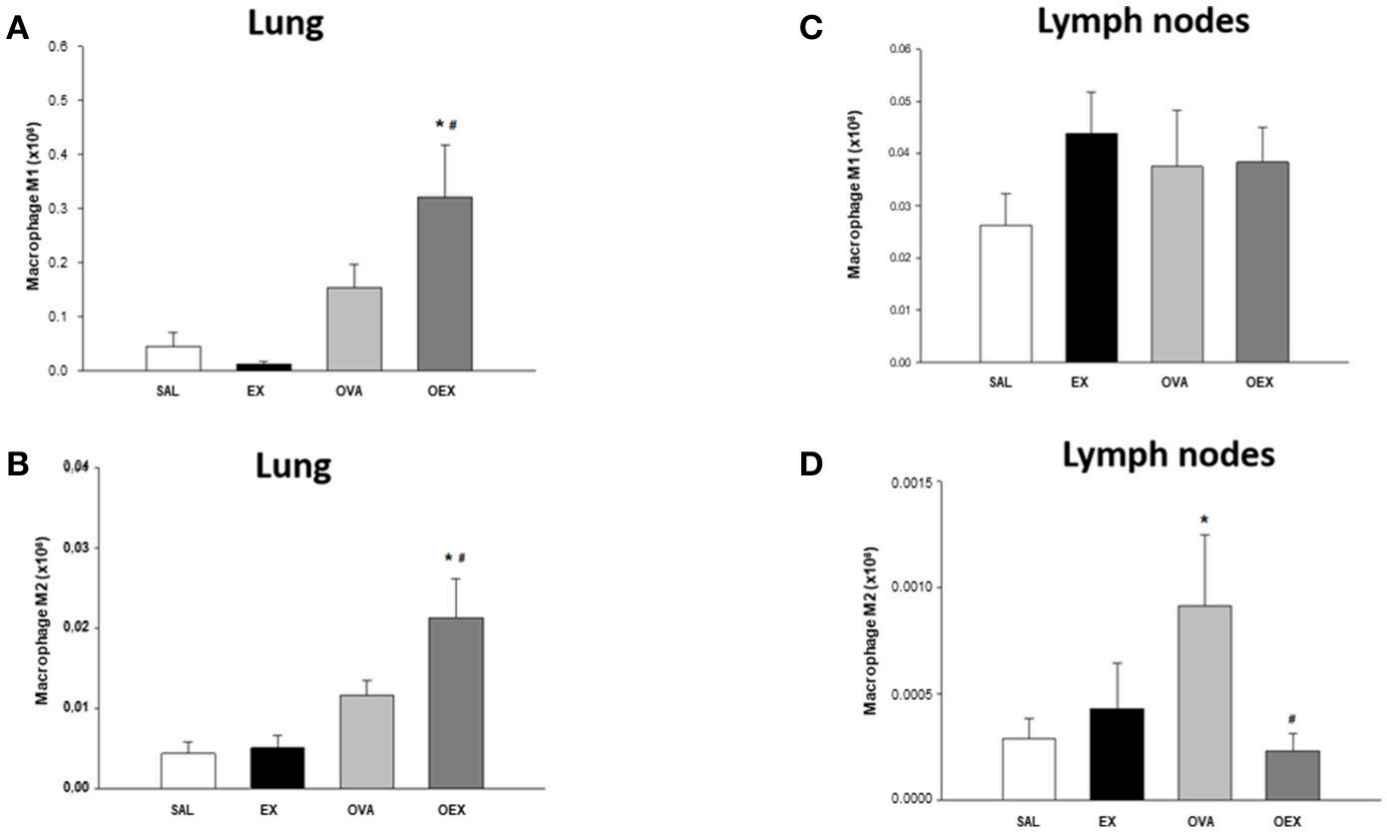
Physical exercise increases the amount of M2 regulatory cells in the lung of sensitized and trained animals as well as M1 but also reduces the presence of M2 in mediastinal lymph nodes. **(A)** The mean values of lung M1 macrophages (±SE) obtained in lung tissue. *p = 0.036 when compared to the EX group and #*p* = 0.020 when compared to the OVA group. **(B)** The mean values of lung M2 macrophages (±SE) obtained in lung tissue. *p = 0.001 compared to the EX group and #*p* = 0.030 when compared to the OVA group. **(C)** The mean values of macrophages M1 (±SE) obtained in lymphoid tissue. **(D)** Mean values of M2 macrophages (±SE) obtained in lymphoid tissue. *p = 0.036 when compared to the EX group and #*p* = 0.020, compared to the OVA group. *N* = 10 per group.

We also evaluated the macrophages present in lung tissue and observed that OVA sensitization and physical exercise increased the levels of total macrophages, both macrophages type 1 (M1) and M2 (Figures 8A,B).

### Physical Exercise Reduces M2 Migration to Draining Lymph Nodes

The evaluation of macrophages presents in the lymph nodes showed that the OVA sensitization increased M2 recruitment and that physical exercise reduced these numbers (Figure 8C). Of note, M1 numbers were not affected by physical exercise or OVA-sensitization (Figure 8D). Taken together with lung analysis, further studies are needed to explain the mechanisms by which physical exercise reduces the presence of M2 in the mediastinal lymph nodes.

### Exercise Leads to an Anti-inflammatory Profile of DCs in the Lymph Nodes

**Figure 9:**
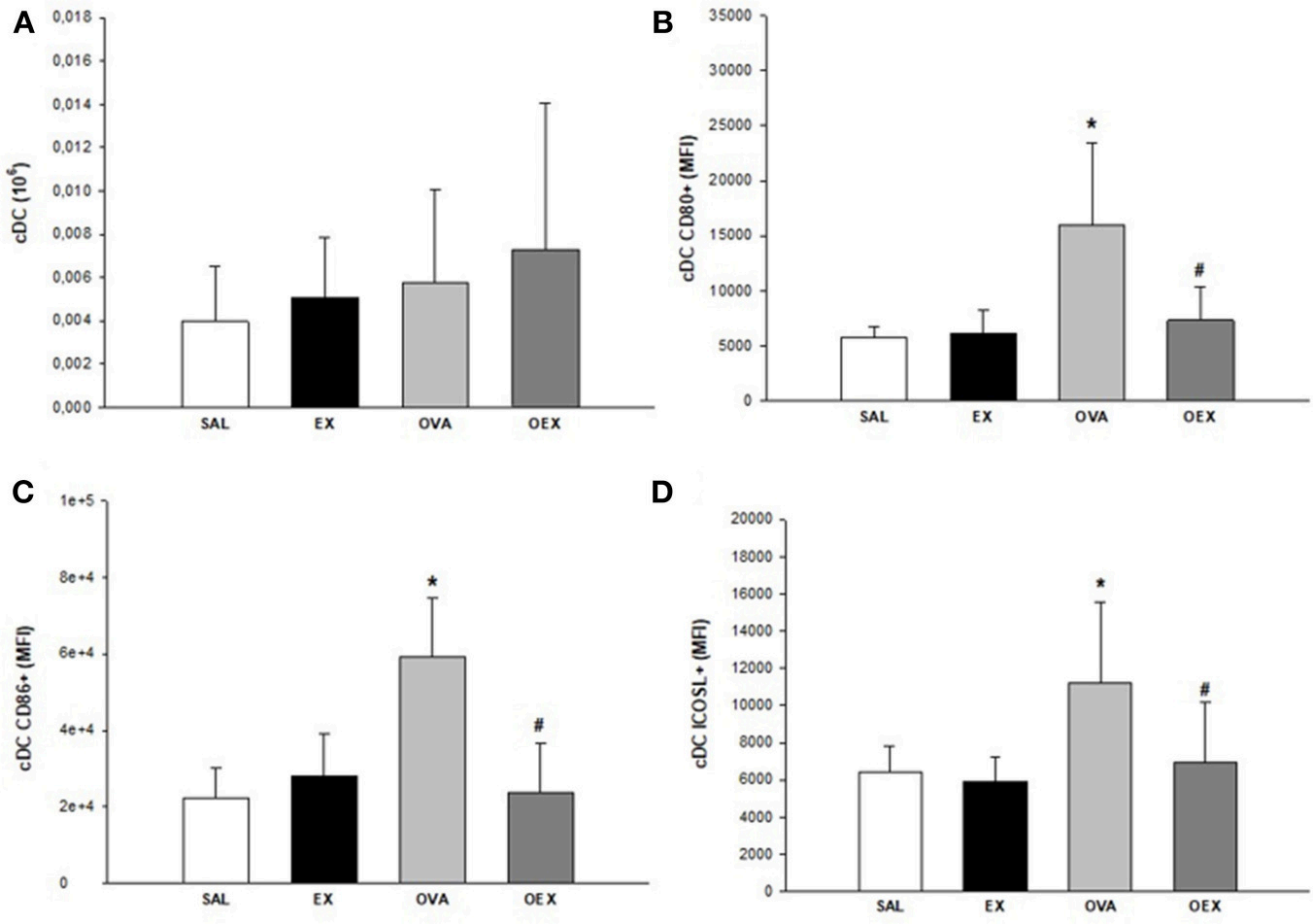
Physical exercise reduces OVA-induced activation in cDCs in lymphoid tissue. **(A)** Quantification of cDCs in lymphoid tissue. Mean values of cDCs (±SE) obtained in lymphoid tissue. **(B)** Sensitization to OVA elevated CD80 expression in cDCs and physical exercise reduced this expression. Mean values of CD80^+^ cDCs (±SE) obtained in lymphoid tissue, *p < 0.001 compared to SAL group, #*p* = 0.001 if compounded to OVA group. **(C)** Sensitization to OVA elevated CD86 expression in cDCs and physical exercise reduced this expression. Mean values of CD86^+^ cDCs (±SE) obtained in lymphoid tissue, *p < 0.001 compared to SAL group, #*p* = 0.001 if compounded to OVA group. **(D)** Sensitization to OVA increased ICOSL expression in cDCs and physical exercise reduced this expression. The mean values of ICOSL^+^ cDCs (±SE) obtained in lymphoid tissue, *p < 0.007 compared to SAL group, #*p* = 0.012 if computed to OVA group. *N* = 10 per group.

**Figure 10:**
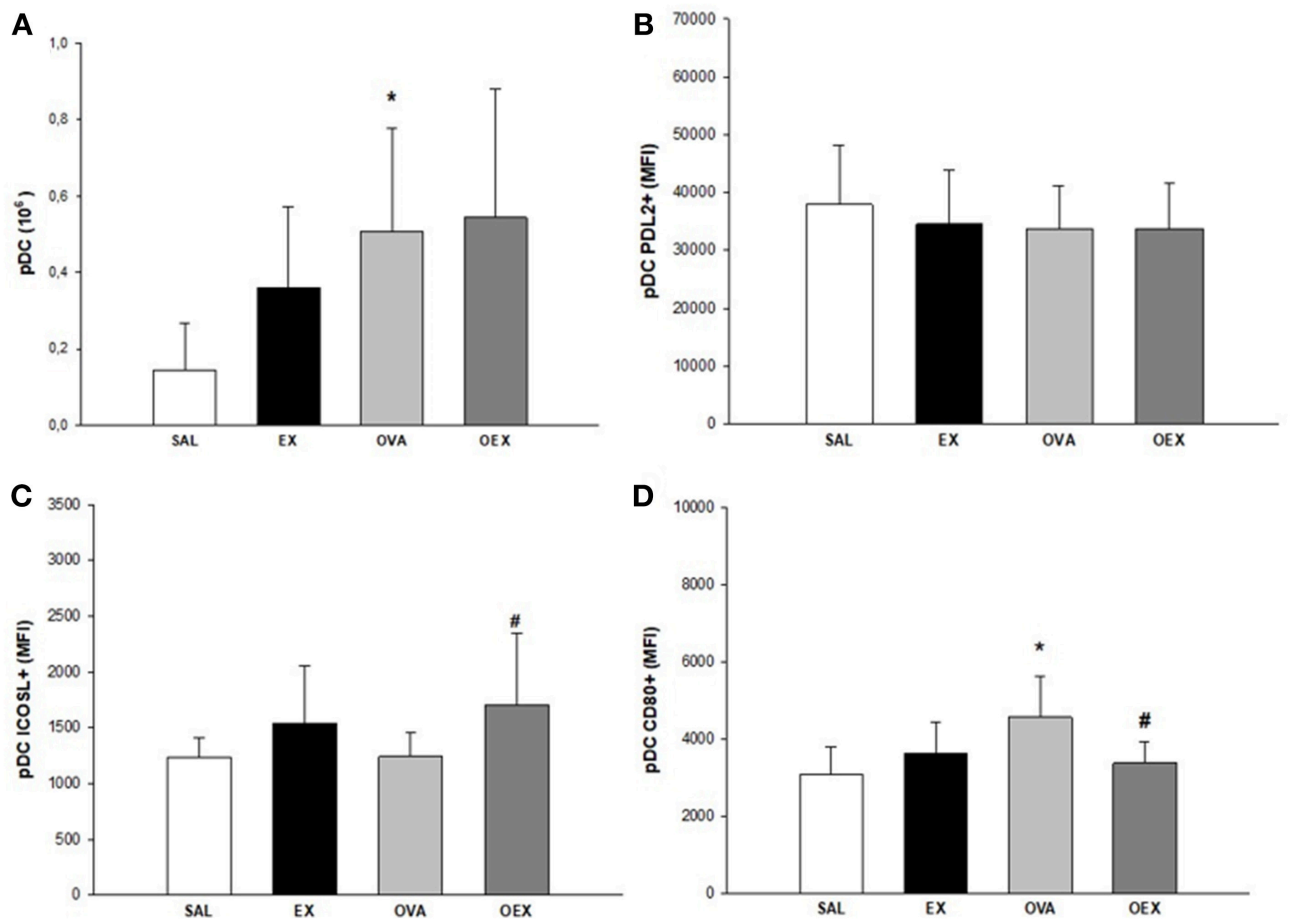
Quantification of pDCs in lymphoid tissue. **(A)** Sensitization to OVA increased numbers of pDCs. The mean values of pDCs (±SE) obtained in lymphoid tissue, *p = 0.024 when compared to SAL group. **(B)** Levels of PDL2 expression in pDCs. The mean values of PDL2^+^ pDCs (±SE) obtained in lymphoid tissue. **(C)** Sensitization to OVA associated with exercise increased the expression of ICOSL^+^ in pDCs. The mean values of ICOSL^+^ pDCs (±SE) obtained in lymphoid tissue, #*p* = 0.029 when compared to the OVA group. **(D)** Sensitization to OVA elevated CD80 expression in pDCs and physical exercise reduced this expression. The mean values of CD80^+^ pDCs (±SE) obtained in lymphoid tissue, *p < 0.005 compared to SAL group, #*p* = 0.021 if compounded to OVA group. *N* = 10 per group.

By evaluating the subtypes of dendritic cells present in the lymph nodes and the expression of co-stimulatory molecules, we found that common DCs (cDCs) from sensitized animals showed increased expression of co-stimulatory molecules such as CD80, CD86, and ICOSL, demonstrating a pro-inflammatory profile of these cells. Physical exercise reverted this activation pattern in cDCs (Figure 9). In addition, there was a significant increase of plasmacytoid DCs (pDCs) in the OVA group that also presented the elevated expression of CD80, indicating a higher activation of these cells. Although the OEX group showed a reduction of CD80 expression by pDCs, these cells were highly activated by increased expression of ICOSL, clearly demonstrating an anti-inflammatory profile in these cells (Figure 10).

## Discussion

The OVA-specific immunoglobulin dosage in the serum of the OVA group demonstrated a significant increase in IgG1, IgG2a, and IgE levels, which were not altered by physical exercise. This suggests that the effects of physical exercise do not act on plasma cells or B cells, which are producers of these immunoglobulins (12).

To evaluate the cellular profile presented in chronic allergic pulmonary inflammation, we quantified and characterized the main cells involved in this inflammatory process (13). We observed that OVA animals showed an increased number of eosinophils present in lung tissue, and physical exercise markedly reduced eosinophilia, indicating that exercise is protective in lung inflammation. The reduction of pulmonary eosinophilia by physical exercise has already been demonstrated by some authors, who demonstrated that there is a reduction in the number of eosinophils in the bronchoalveolar lavage and pulmonary tissue of asthmatic animals following physical exercise (14, 15).

Elevated levels of IL-4 and IL-6 were detected in the OVA group, and interestingly, moderate exercise reduced the levels of these cytokines in the lung of sensitized mice. Some studies have shown that animals sensitized to OVA show a significant increase in the levels of these cytokines and that there is a reduction when the animals undergo a exercise protocol, corroborating our findings (7, 15–17). We could also compare the reduction of the IL-4 levels with lower lung eosinophilia once there is evidence, suggesting that this cytokine increases airway eosinophilia (18).

Physical exercise promotes anti-inflammatory responses in a lung inflammation model by increasing anti-inflammatory cytokines such as IL-10 and IL1-Ra (3, 19, 20). In addition to the increased levels of IL-10, we also noted an increase in TGF-β1 levels in lung homogenate from groups that underwent physical exercise, indicating that there is another regulatory cytokine co-participating in the anti-inflammatory process of physical exercise. *In vitro* experiments have shown that TGF-β1 has a regulatory function in both innate and adaptive immune cell function (21). Furthermore, TGF-β1 is associated with suppressor T cell immune function in that it suppresses immune responses through inhibition of inflammatory cell function and promotion of Treg production, as a Treg inducer (22). Of note, Tregs are the main source for TGF-β and IL-10. We observed an increase in Treg numbers in the lungs. In addition, the high expression of CD25 and LAP reinforces the hypothesis that these cells are regulating the lung inflammation. LAP is an important marker of TGF-β excretion in Tregs. TGF-β is produced as an inactive latent complex made up of LAP (23).

We observed an increased influx of lymphocytes, both of CD4 and CD8 T cells, in the lungs and lymph nodes in the OEX group. CD8 T cells show immunoregulatory/immunosuppressive capacities in a CD4 T cell-associated inflammatory disease such as asthma (24, 25). Activated CD8 T cells can generate a regulatory microenvironment through adhesion molecules and release of cytokines, indicating a potential to induce regulation in DCs and finally, activation and proliferation of CD4 T cells. In this same context, DCs play a central role in the activation and proliferation of CD4 T cells. It has also been shown that CD8 T cells have the capacity to negatively regulate splenic DC phenotypes, such as the expression of CD86, CD80, and MHC-II (26). Of note, it is known that the tolerogenic functions of DCs are closely related to immunosuppressive cytokines such as TGF-β and IL-10 (26, 27). In the lymph nodes, we observed a decrease of CD80, CD86, and ICOSL expression in cDCs of trained and sensitized mice when compared to the sensitized and challenged group. When we examined pDC in the same compartment, we noticed the increase of pDCs in mice only OVA-sensitized, whereas the exercise increased ICOSL expression and decreased CD80. *In vivo* and *in vitro* studies have both clearly shown that pDCs can stimulate the induction of Treg cells, possibly in an ICOSL-dependent way (28). Taken together, these data indicates an anti-inflammatory pattern induced by exercise on DCs. Increased levels of IL-10, observed both in the sensitized and trained animal samples, are linked with decreased expression of the co-stimulatory molecules CD80 and CD86. Studies have already shown that IL-10 has the ability to decrease the expression of these co-stimulatory molecules, such as MHC-II and, therefore, reduce the DC pro-inflammatory phenotype (29).

We also analyzed macrophages in the lung tissue. In the OEX group, the increase in the amount of M2 macrophages could be directly linked to the increase in IL-10 measured in the lung tissue (27). Interestingly, the reduction in the numbers of M2 macrophages in the lymph nodes of the sensitized and trained mice (OEX) indicates the interference of physical exercise in the migration of these cells. Additional studies must be performed to clarify this issue.

Thus, we conclude that physical exercise induces an increase of M2 macrophages in the lungs, in addition to an anti-inflammatory profile of DCs and an increase of Treg cells in OVA-sensitized and challenged mice. These findings help to better understand some of the mechanisms underlying how exercise regulates chronic allergic inflammation.

## Ethics Statement

The Ethics Committee of the School of Medicine of the University of São Paulo approved all animal experiments (Protocol number 067/16). Male Balb/c mice (6–8 weeks old) were purchased from the University of São Paulo (São Paulo, Brazil) and maintained as described in the Guide for the Care and Use of Laboratory Animals—NIH (8).

## Author Contributions

PF performed the experiments, analyzed all data, and drafted the manuscript text. LO and MS helped plan the flow cytometry experiments and LO performed them. TB helped with data analysis and manuscript writing. CO helped with the experimental exercise protocol. FA-C designed the study, reviewed the data, and helped write the manuscript text.

### Conflict of Interest Statement

The authors declare that the research was conducted in the absence of any commercial or financial relationships that could be construed as a potential conflict of interest.
