# Supplementary material for: Physical Exercise Induces Immunoregulation of TREG, M2, and pDCs in a Lung Allergic Inflammation Model
Source: Front Immunol. 2019 May 16;10:854. doi: 10.3389/fimmu.2019.00854 (PMC6532549; doi:10.3389/fimmu.2019.00854)
Supplement: Supplementary file 1 [file Data_Sheet_1.docx]

**SUPPLEMENTARY MATERIAL**


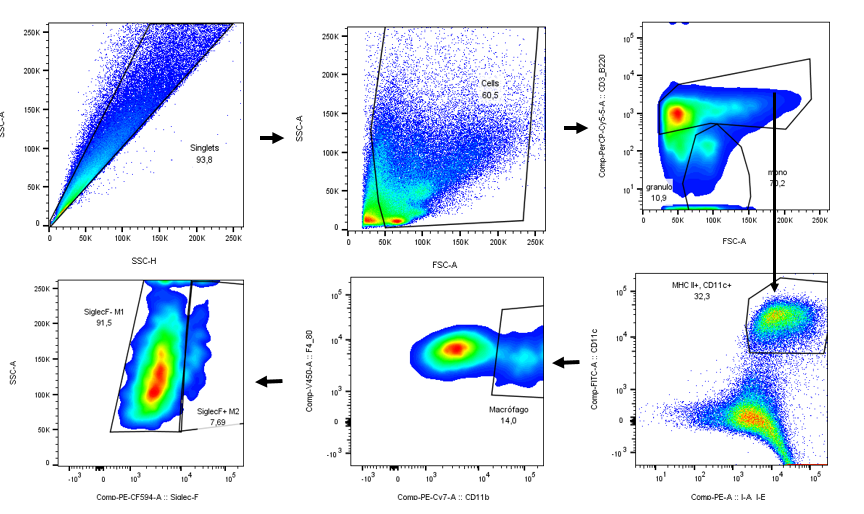
 **Supplementary Figure 1 - Analysis strategy for macrophages.** Macrophages are considered FSC-A high and CD3/B220 intermediate/low. Type 1 macrophages were considered CD11c positive, MHC II positive and Siglec-F negative. Type 2 macrophages were considered CD11c positive, MHC II positive and Siglec-F positive.

**
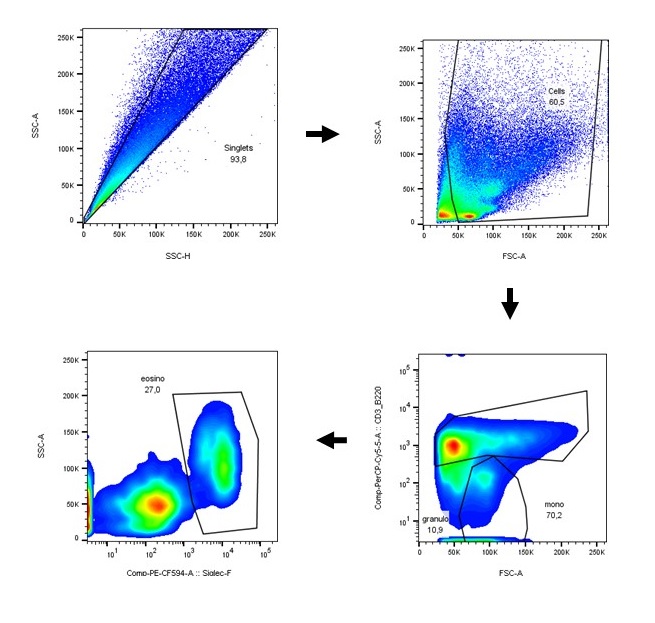
**

**Supplementary Figure 2 - Analysis strategy for eosinophils in lung homogenate.** Granulocytes are considered FSC-A low and CD3/B220 negative. Then, eosinophils were considered as Siglec-F positive cells.


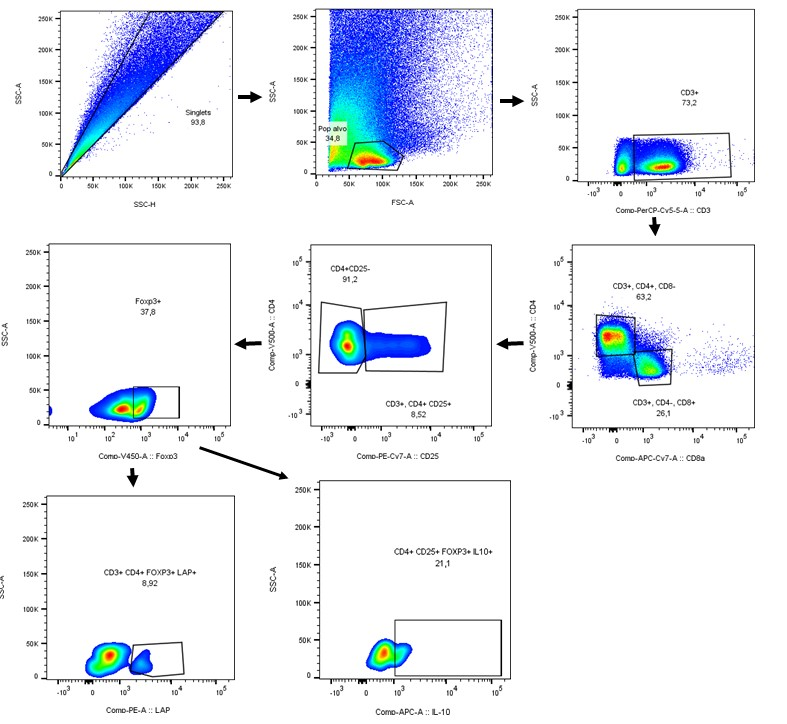


**Figure 3** **- Analysis strategy for T regs in the lung.** All lymphocytes were considered positive for CD3. CD4+ T lymphocytes were considered activated when were positive for CD69. T regs were considered CD3, CD4, CD25 and Foxp3 positive. Then we gated Treg that expressed LAP, an indicator of TGF-β production, or IL-10.


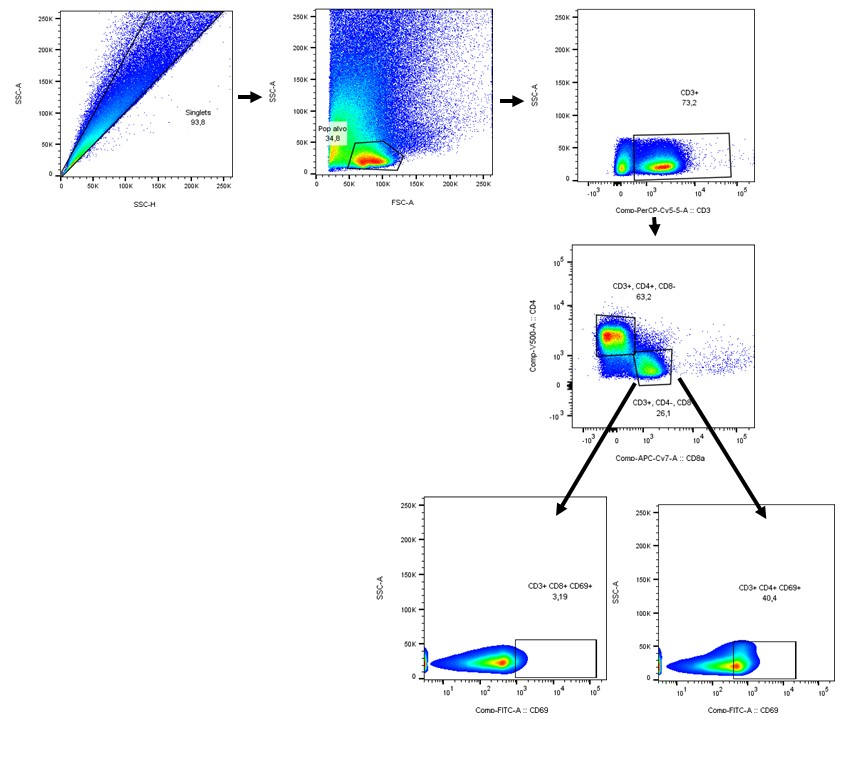
 **Figure 4** - **Analysis strategy for CD4+ and CD8+ T lymphocytes and activation of CD8+ T lymphocytes by expression of CD69 in lung.** All lymphocytes were considered positive for CD3. CD4+ or CD8+ T lymphocytes were considered activated when they were positive for CD69.


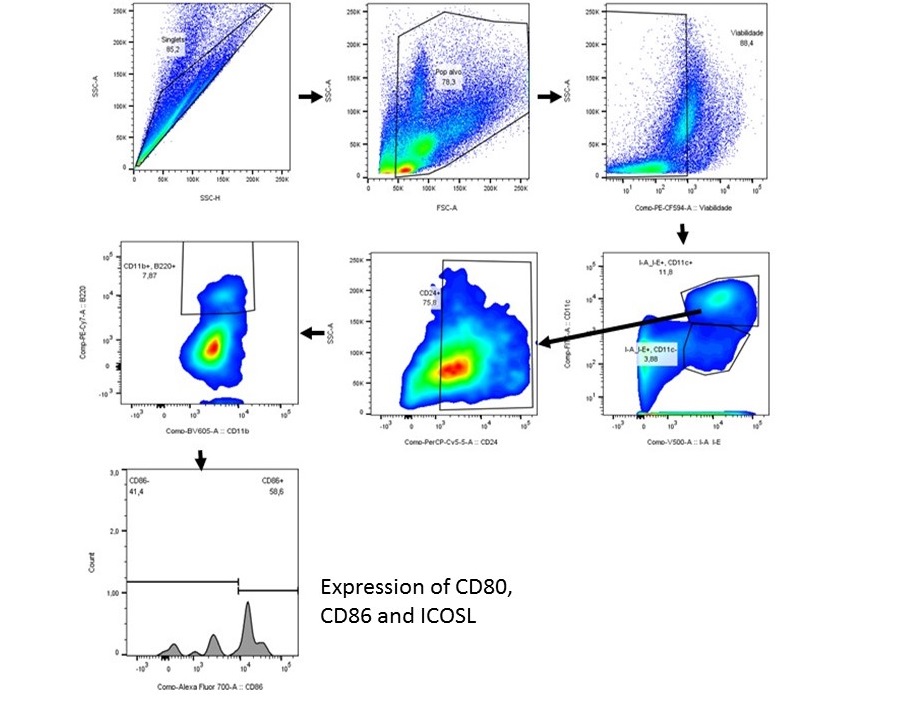


**Figure 5** - **Analysis strategy for common dendritic cells (cDCs) and activation by expression of CD80 in lung.** cDCs were considered CD11c and MHC II high, positive for CD24, B220 and CD11b high. Cells were considered activated when positive for CD80.


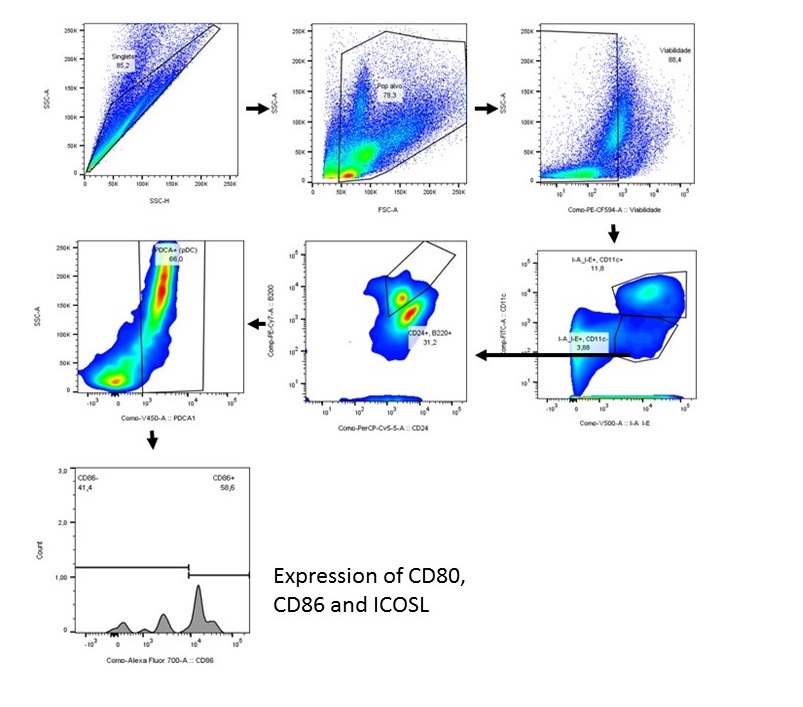


**Figure 6** - **Analysis strategy for plasmacytoid DCs (pDCs) and activation by expression of CD86, ICOSL and PDL2 in lung.** pDCs were considered CD11c negative or low, MHC II low, positive for B220, CD24 and PDCA1. Cells were considered activated when positive for CD86, or ICOSL or PDL2.
